# Distinguish between Stochastic and Chaotic Signals by a Local Structure-Based Entropy

**DOI:** 10.3390/e24121752

**Published:** 2022-11-30

**Authors:** Zelin Zhang, Jun Wu, Yufeng Chen, Ji Wang, Jinyu Xu

**Affiliations:** 1 School of Mathematics, Physics and Optoelectronic Engineering, Hubei University of Automotive Technology, Shiyan 442002, China; 2 Hubei Key Laboratory of Applied Mathematics, Hubei University, Wuhan 430061, China; 3 School of Electrical and Information Engineering, Hubei University of Automotive Technology, Shiyan 442002, China; 4 School of Liberal Arts and Humanities, Sichuan Vocational College of Finance and Economics, Chengdu 610101, China

**Keywords:** multivariate time series, information entropy, chaotic sequence, random signal, machinery fault diagnose

## Abstract

As a measure of complexity, information entropy is frequently used to categorize time series, such as machinery failure diagnostics, biological signal identification, etc., and is thought of as a characteristic of dynamic systems. Many entropies, however, are ineffective for multivariate scenarios due to correlations. In this paper, we propose a local structure entropy (LSE) based on the idea of a recurrence network. Given certain tolerance and scales, LSE values can distinguish multivariate chaotic sequences between stochastic signals. Three financial market indices are used to evaluate the proposed LSE. The results show that the $LSE^{FSTE100}$ and $LSE^{S\&P500}$ are higher than $LSE^{SZI}$, which indicates that the European and American stock markets are more sophisticated than the Chinese stock market. Additionally, using decision trees as the classifiers, LSE is employed to detect bearing faults. LSE performs higher on recognition accuracy when compared to permutation entropy.

## 1. Introduction

The local structure and its integration are crucial components in defining the vast system as a whole. For instance, the personal abilities of football players on the field and their teamwork play key roles in a competition [1], or the seismic capacities of buildings rely on materials and frame structures [2]. However, people tend to use local structures to identify objects in machine learning work [3,4]. Numerous examples can be found in feature selection [5,6], pattern recognition [7,8,9], and so on. Is it possible that a local structure is enough to accomplish identification? If it is possible, it means that other methods, such as global–local structure-based learning [10], are not necessary. So, no one can deny that it depends on specific issues. People tend to agree that only when local structures contain enough information for judging can the local structure-based strategies take effect. Therefore, finding the amount of information contained in the local structures of samples is important for further exploration.

In the time series analysis field, entropies were used to reflect the amount of information contained in observed data. The first one can be traced back to Shannon entropy [11], which is defined as,

(1) $$H\left(X\right)=-\sum_{x\in X}p\left(x\right)\ln\left(p\left(x\right)\right)$$

where *X* is a random variable and p(x) is its probability density. For a time series, Equation (1) is valid under the assumption of stationarity despite whether *X* is univariate or multivariate. Interested readers can refer to other entropies based on Equation (1) for univariate cases, such as sample entropy [12,13], multiscale entropy [14,15], permutation entropy (PE) [16,17,18], etc. These methods have one thing in common—the observed time series is embedded into a phase space in order to reflect the autocorrelation. However, the above-mentioned entropies, except for PE, cannot be directly generalized to multivariate cases. With regard to PE, although it naturally provides a multivariate version [19,20,21], it does not reflect correlations between components. Although the Kullback–Leibler entropy [22] is a classical tool to assess the correlation between two components, it is asymmetrical and cannot be employed on a time series whose dimension is greater than 2. In general, correlations between different components together with autocorrelation in every component, are considered double-edged swords, which provide positive affection where the times series can be predicted, as well as a negative influence, where it is difficult to measure the complexity of a multi-dimensional time series. Researchers managed to introduce other entropies to characterize multivariate sequences, such as matrix-based entropy [23,24], multiscale entropy [25,26,27], and estimation methods [28]. For instance, Azami proposed a refined composite multivariate multiscale fuzzy entropy that demonstrates long-range, within, or cross-channel correlations [29]. Han proposed a multivariate multi-scale weighted permutation entropy to illustrate oil–water two-phase flow instability [30]. Mao constructed a complexity–entropy causality plane based on the normalized Shannon entropy and multivariate permutation entropy, thus characterizing various chaotic systems [31]. With the complex network method, Shang put forward another complexity–entropy causality plane via multiscale entropy and the degree distribution of the vector visual graph to measure the complexity of the stock index [32]. The above-mentioned methods have at least one of the following drawbacks: (1) the observed sample numbers need to be large, otherwise the results are not reliable; (2) the computing times are long, so they cannot satisfy the real-time requirements of the application; (3) focusing on autocorrelation but neglecting relationships between components. To illuminate those shortcomings, we plan to use a complex network-like approach to represent the local structure.

In the past decades, we have witnessed the development of complex network science [33,34,35], which is utilized to present complex systems such as climate dynamics [36] and human language [37]. Meanwhile, it is regarded as a newly advanced tool for time series analysis, such as for univariate cases [38,39,40] and multivariate cases [41]. Interested readers can find more information in the review articles [42,43]. These complex network-based methods can be divided into three classes: visibility graph (VG) [44,45], recurrence net (RN) [46,47], and transition net (TN) [48,49]. For VG, a vertex is obtained by the linear relationship contained in fragments. For TN, a vertex is usually generated by certain coarse-graining strategies. Links between pairs of adjacent vertexes in VG or TN are naturally produced according to the order. However, RN is quite different. Its edges are bridged by the similarities of observed values. The validities of these methods also indicate that local structures together with their combination forms could represent essential characteristics of a complex system. Inspired by the above means, we propose a local structure-based entropy (LSE) for analyzing a multivariate time series. This can be regarded as a multivariate version of the Shannon Entropy defined on a discrete distribution of the local structure; the recurrence of the observed vector is employed to represent local structure information. In fact, RN is constructed by the similarity between all observed data. If we just consider the reappearances of the initial state in the sliding window, then its distribution can be viewed as a manifestation of local structure.

The rest of this paper is organized as follows. Section 2 introduces the process of computing LSE; Section 3 tests LSE on the fractional-order chaotic time series; LSE is applied to the stock market index and bearing fault signals; finally, we draw our conclusions in Section 5.

## 2. Method

In [50], Donner introduced the construction of RN, which takes individual values as vertices and indicators of recurrences as edges. Here, a pair of states whose values are close enough can be regarded as recurrences. Thus, RN represents the reappearance of system states over a long period. It is natural for one to (hope to) use the recurrent numbers of the initial states in sliding windows of different lengths to characterize the complexities of the systems.

Let $x_{t}={\left(x_{1t},x_{2t},\ldots ,x_{mt}\right)}_{t\leq N}^{T}$ be an *m*-dimensional time series. τ and *i* are integers with i+τ−1≤N. Extract fragment $Y_{i}$ from $x_{t}$ by

(2) $$Y_{i}=\left(\begin{array}{ccc} x_{1,i} & \ldots & x_{1,(i+\tau -1)}\\ \vdots & \vdots & \vdots \\ x_{m,i} & \ldots & x_{m,(i+\tau -1)} \end{array}\right)$$

**Figure 1:**
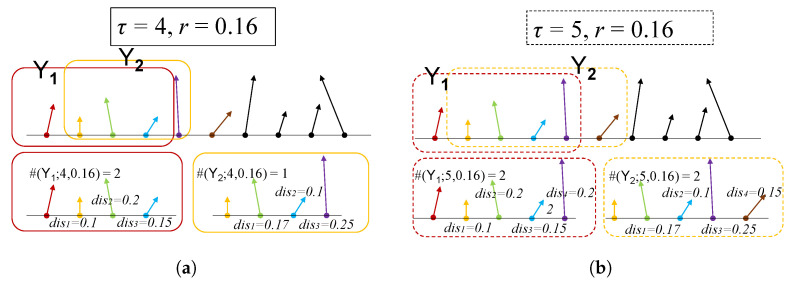
Schematic diagram of $\#(Y_{i};\tau ,r)$ procedure. (**a**) tolerance r=0.16, scale τ=4, (**b**) r=0.16, τ=5.

Let j∈{0,1,2,…,τ−1}, $y_{j}^{i}$ denotes the (j+1)th column vector of $Y_{i}$. Define

(3) $$dis_{j}=\left|y_{0}^{i},y_{1+j}^{i}\right|$$

where |·,·| is a distance in $R^{m}$ (in this paper, we use the Euclidean distance). Given a tolerant threshold *r* and the number of *j*, such that $dis_{j}\leq r$ is called the recurrent number of $Y_{i}$ with the scale τ, and the tolerance *r* is denoted by $\#(Y_{i};\tau ,r)$. That is,

(4) $$\#\left(Y_{i};\tau ,r\right)=\sum_{j\in \{1,2,\ldots ,\tau -1\}}\mathrm{sign}\left(dis_{j}\leq r\right)$$

where sign(·) is the indicator function. Two examples of the above procedure are demonstrated in Figure 1.

Now, the LSE of $x_{t}$ under scale τ and tolerance *r* is defined as

(5) $$LSE\left(x_{t};\tau ,r\right)=\sum g\left(\#\left(Y_{i};\tau ,r\right)\right)\ln\left(g\left(\#\left(Y_{i};\tau ,r\right)\right)\right)$$

where $g\left(\cdot \right)=p\left(\cdot \right)/a_{\tau ,r}$, p(·) represents the density function, and $a_{\tau ,r}$ is the average of $\#(Y_{i};\tau ,r)$.

**NOTE 1** The value of LSE depends on the selection of τ and *r*. When their ranges are given, they can be chosen by the maximal LSE. Namely,

(6) $$\left(\tau ,r\right)=\underset{\tau ,r}{\mathrm{argmax}}\;LSE\left(x_{t};\tau ,r\right)$$

In general, the range of τ can be decided by the length of the time series, such as 1/100 to 1/10 of the raw data. The range of *r* can be selected by referring to the standard deviation of the multivariate standard Gaussian series. See Appendix A.1.

**NOTE 2** Given a set of values for τ and *r*, respectively, such as $\tau \in \{\tau_{1},\tau_{2},\ldots ,\tau_{q}\}$ and $r\in \{r_{1},r_{2},\ldots ,r_{s}\}$, the values of $LSE(x_{t};\tau_{i},r_{j})$ form a matrix, which presents the variation of the complexity of $x_{t}$ over different scales and tolerances. Therefore, it could be used to characterize $x_{t}$.

**NOTE 3** We adopt two strategies for further exploration. To designate the corresponding LSE values, we utilize the variables $LSE_{A}$ and $LSE_{B}$. METHOD A: normalize each component of $x_{t}$ and specify a tolerance range as described above, which leads to $LSE_{A}$ being immune to linear transformations; METHOD B: do not normalize $x_{t}$ but set tolerance range by the total of component variances, thus taking into account the extents of various channel volatilities. Broadly speaking, $LSE_{A}$ and $LSE_{B}$ are not equivalent, and they can be seen as two different ways of describing the initial sequence. See Appendix A.2 for more information.

## 3. Numerical Simulations

In this section, we test LSE on several multivariate deterministic and stochastic signals. At first, we analyze fractional-order chaotic systems and random series and find out ranges of τ and *r* in Section 3.1, then we test LSE on integer-order, fractional-order chaotic systems and random sequences in Section 3.2.

### 3.1. Fractional-Order Chaotic Systems and Random Series

Derivatives and integrals of fractional orders are employed to describe objects with memory properties, such as power law nonlocality or long-range dependence [51] and, thus, can model real-world systems more accurately than the classical integer calculus. Many fractional-order dynamical systems [52,53,54] with total order of less than three can exhibit chaos while continuous nonlinear systems with the total order of less than three cannot under the concept of the usual integer order. In this section, we simulate three multivariate fractional-order dynamical models and three random signals to test LSE.

For many classes of functions, the three most well-known fractional-order derivatives (the Grünwald–Letnikov (GL), Riemann–Liouville, and Caputo) are equivalent under some conditions [55]. Here, we use the GL definition, i.e.,

(7) $$D_{t}^{\alpha }f\left(t\right)=\lim_{h\to 0}\frac{1}{h^{\alpha }}\sum_{j=0}^{\infty }{\left(-1\right)}^{j}\left(\begin{array}{c} \alpha \\ j \end{array}\right)f\left(t-jh\right)$$

Then, for the fractional-order differential equation

(8) $${}_{a}D_{t}^{\alpha }y\left(t\right)=f\left(y\left(t\right),t\right)$$

a general numerical solution has the form of [56]

(9) $$y\left(t_{k}\right)=f\left(y\left(t_{k}\right),t_{k}\right)h^{\alpha }-\sum_{j=v}^{k}c_{j}^{\left(\alpha \right)}y\left(t_{k-j}\right)$$

where

(10) $$c_{0}^{\left(\alpha \right)}=1,\;c_{j}^{\left(\alpha \right)}=\left(1-\frac{1+\alpha }{j}\right)c_{j-1}^{\left(\alpha \right)}$$

**Figure 2:**
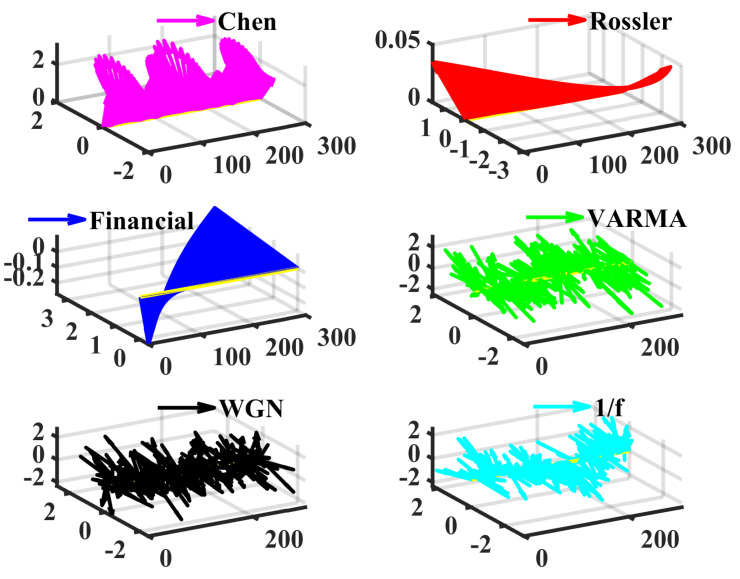
Examples of the chaotic and stochastic series. In fact, we view the multivariate time as a vector sequence. The first axis is the time, (*t*,0,0) is regarded as the original point for plotting the vectors (x(t),y(t),z(t)). The length is 300, and the time step is 0.005. Note that the series of Rössler and the financial systems appear to be very simple variations.

Then, several different dynamic systems, including the fractional order chaotic system, multivariate vector autoregression moving-average process (VARMA), white Gaussian noise (WGN), and 1/f noise, are generated and then analyzed by LSE to showcase its effectiveness. The method in [56] is adopted to generate numerical solutions of Equations (11) to (13); they are chaotic time series, which are cross-validated with the largest Lyapunov exponent [54]. The simulation timespan is 0:0.005:30. (Start at 0, end at 30, the step is 0.005). To avoid the influences of the initial values, the first 10% of data are discarded and the sampled series (500×3, 500 simulated three-dimensional vectors) start from a random position. Figure 2 displays examples of these signals.

1. Fractional-order Chen system [52]
  (11) $$\left\{\begin{array}{c} D^{\alpha_{1}}x=a\left(y-x\right)\\ D^{\alpha_{2}}y=dx-xz+cy\\ D^{\alpha_{3}}z=xy-bz \end{array}\right.$$
  $\alpha_{1}=\alpha_{2}=\alpha_{3}=0.9$, a=35, b=3, c=28, d=c−a=−7, initial values $\left(x_{0},y_{0},z_{0}\right)=\left(-9,-5,14\right)$.
2. Fractional-order Rössler system [53]
  (12) $$\left\{\begin{array}{c} D^{\alpha_{1}}x=y-z\\ D^{\alpha_{2}}y=x+ay\\ D^{\alpha_{3}}z=b+z\left(x-c\right) \end{array}\right.$$
  $\alpha_{1}=0.9$, $\alpha_{2}=0.85$, $\alpha_{3}=0.95$, a=0.5, b=0.2, c=10, $\left(x_{0},y_{0},z_{0}\right)=\left(0.5,1.5,0.1\right)$.
3. Fractional-order financial system [54]
  (13) $$\left\{\begin{array}{c} D^{\alpha_{1}}x=z+\left(y-a\right)x\\ D^{\alpha_{2}}y=1-by-x^{2}\\ D^{\alpha_{3}}z=-x-cz \end{array}\right.$$
  $\alpha_{1}=1$, $\alpha_{2}=0.95$, $\alpha_{3}=0.99$, a=1, b=0.1, c=1, $\left(x_{0},y_{0},z_{0}\right)=\left(2,-1,1\right)$.
4. Multivariate vector autoregression moving-average process
  (14) $$y_{t}=c+\sum_{j=1}^{p}\Phi_{j}y_{t-j}+\sum_{k=1}^{q}\Theta_{k}\epsilon_{t-k}+\epsilon_{t}$$
  $\Phi_{1}=\left(\begin{array}{ccc} 0.2 & -0.1 & 0\\ 0.1 & 0.2 & 0.05\\ 0 & 0.1 & 0.3 \end{array}\right)$, $\Phi_{2}=\left(\begin{array}{ccc} 0.3 & 0 & 0\\ 0.1 & 0.4 & 0.1\\ 0 & 0 & 0.2 \end{array}\right)$, $\Phi_{3}=\left(\begin{array}{ccc} 0.4 & 0.1 & -0.1\\ 0.2 & -0.5 & 0\\ 0.05 & 0.05 & 0.2 \end{array}\right)$, $\Theta_{1}=\left(\begin{array}{ccc} 0.2 & 0 & 0\\ 0 & 0.2 & 0\\ 0 & 0 & 0.2 \end{array}\right)$, $\Theta_{2}=\left(\begin{array}{ccc} 0.3 & 0.2 & 0.1\\ 0.2 & 0.4 & 0\\ 0.1 & 0 & 0.5 \end{array}\right)$, $c={\left(0.05,0,-0.05\right)}^{T}$, covariance matrix is the unit matrix, ϵ is a standard Gaussian White noise, and the length equals to the above numerical solutions. This procedure is completed by the ARMA2AR and VARMA function of MATLAB2020b.
5. White Gaussian noise
  We use the NORMRND function of MATLAB2020b to simulate WGN (500 × 3). Its components are independent with zero mean and unit standard deviation.
6. 1/f noise
  On the basis of the algorithm in [57], the procedure of generating 1/f noise consists of three basic steps: (i) simulating white noise whose length is 1500, we obtain $y_{0}$; (ii) DFT (discrete Fourier transformation) on $y_{0}$, multiplied by $f^{-\frac{1}{2}}$ and symmetrized for a real function, then IDFT (inverse discrete Fourier transformation), adjust the mean and standard deviations, yielding $y_{1}$; (iii) resize $y_{0}$ and $y_{1}$ to 500×3 matrix; finally, the 1/f noise series is composed as $\left[y_{0}\left(:,1\right),y_{1}\left(:,2\right),\frac{1}{2}\left(y_{0}\left(:,3\right)+y_{1}\left(:,3\right)\right)\right]$.

**Figure 3:**
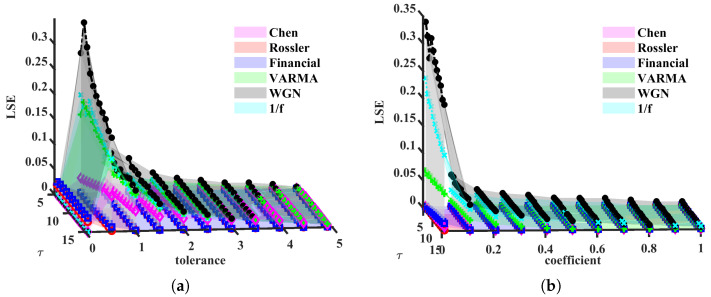
LSE(Local structure based entropy) values, as functions of τ and *r*. (**a**) METHOD A, (**b**) METHOD B.

Now, we set the simulation parameters as follows: τ∈{5,6,…,15}, $r\in \left\{0.05:0.041:0.5\right\}\times \sqrt{24}$ for METHOD A, r∈{0.05:0.041:0.5}×Σ for METHOD B, where $\Sigma =\sum_{i=1}^{3}\sigma_{i}^{4}$ and $\sigma_{i}$ is the standard deviation of *i*th component. More details can be found in Appendix A.2. LSE values are shown in Figure 3.

**Figure 4:**
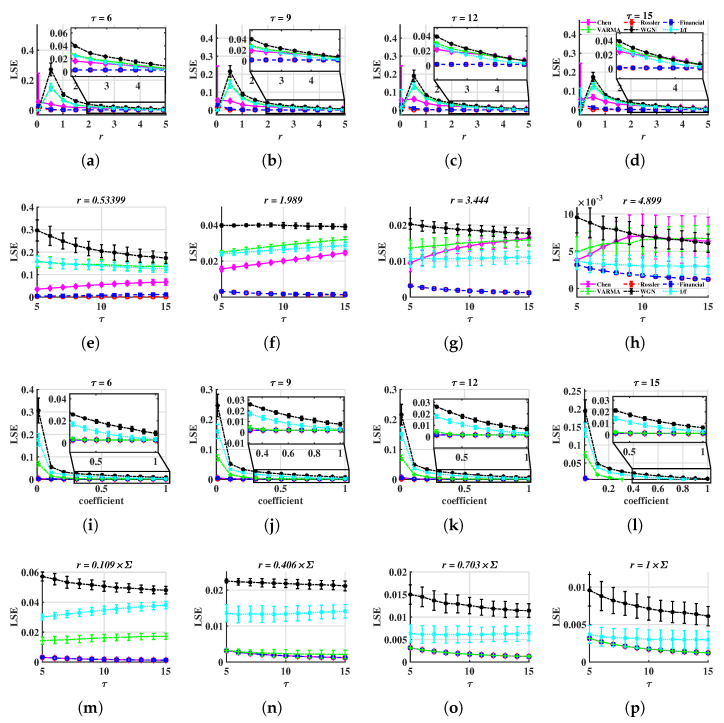
LSE values, as functions of τ or *r*. The time step is 0.005 for Equations (11) to (13). The marker presents the average and the error bar presents the standard deviation of LSE for fixed τ and *r*. (**a**–**h**) METHOD A, (**i**–**p**) METHOD B.

From Figure 3, we found both METHOD A and METHOD B can distinguish multivariate chaotic signals from stochastic ones. For a fixed (τ,r), the LSE of random time series is higher than that of a chaotic one. As shown in Figure 3a, WGN has the most complex signals, the 1/f noise and VARMA series appear to have similar trends, and the other three chaotic series lie under 0.1. They keep the same orders for most (τ,r). In Figure 3b, LSE values are significantly different and hardly overlap, except for Chen and Rössler systems. The reason these two systems are indistinguishable can be because the range of coefficients of *r* is rather small. Next, we change the lower and upper bounds to 0.01 and 1. Moreover, the above simulation will be repeated 100 times (100 trajectories for each system) to check the robustness of LSE. In each simulation, we add small disturbances to initial values ($Disturb_{initial}=0.3\times \epsilon$)). Time series generated from Equations (11) to (13) are still chaos with these disturbances; refer to [56]. Note that the intersections of LSE surfaces and $\tau =\tau_{i}$ (or $r=r_{i}$), other than surfaces themselves, are utilized to verify the validity of our methods. Subjecting to the space, only part of the results are demonstrated, see Figure 4 and Figure 6.

In Figure 4, the LSE of random series is higher than that of chaotic ones, thus we believe LSE can be treated as an efficient tool to characterize multivariate time series. When τ is fixed, LSE values of different dynamics show their own features. In Figure 4a–d, LSE values of chaotic systems show similar trends where $LSE_{A}^{chaos}$ oscillates heavily when $r=r_{1}=0.01\times \sqrt{24}$. Then, $LSE_{A}^{chaos}$ decreases as tolerance increases. For $LSE_{A}^{random}$, they reach their highest values at $r_{2}$ and then keep reducing as *r* increases. However, for METHOD B, all LSE values are decreasing, see Figure 4i–l. When *r* is fixed, except for the Rössler and Financial systems, other series can be distinguished by LSE values over different scales. For instance, in Figure 4e, $LSE_{A}^{chen}<0.08$ but $LSE_{A}^{random}>0.1$, in Figure 4f, $LSE_{A}^{WGN}$ lie at the highest level, and Figure 4g,h,m,n, $LSE^{1/f}$ locate higher than $LSE^{VARMA}$.

Compare METHOD A with METHOD B, when *r* is small, the later performs better at distinguishing multivariate stochastic series from chaotic ones, as well as more stable LSE values for chaos (basically the same). For instance, all $LSE^{chaos}$ are overlapped and smaller than $LSE^{random}$ when the coefficient is 0.109 in Figure 4m. However, in Figure 4e,f, $LSE_{A}^{Chen}$ is close to $LSE_{A}^{random}$. Moreover, the former shows better discrimination on chaotic systems. This is because of the standardization process of METHOD A, which weights all components at an equal level. It may amplify the LSE if the system’s variation is caused by only one or a small group of components.

Based on the simulation findings mentioned above, the following reference range of parameters can be provided:

- 5≤τ≤15 is acceptable. In Figure 4a–d and in Figure 4i–l, when *r* is taken appropriately, these curves have essentially the same shape, making it possible to distinguish between different signals. This suggests that the distinction is not dependent on the value of τ, and the corresponding time complexity can be taken into consideration when designing this parameter.
- For METHOD A, 0.5≤r≤2. For METHOD B, 0.1≤coefficient≤0.4. For example, when r=0.53399, $LSE^{chaos}$ is less than 0.1 but $LSE^{random}$ is higher than 0.1. Therefore, the stochastic sequence can be distinguished from chaotic ones. See Figure 4e. If *r* is too small, LSE has a large standard deviation, as is shown in Figure 4a,i. If *r* is too large, $LSE^{chaos}$ and $LSE^{random}$ are overlapped, thus, it is difficult to tell apart various signals. See Figure 4g,o.

**Figure 5:**
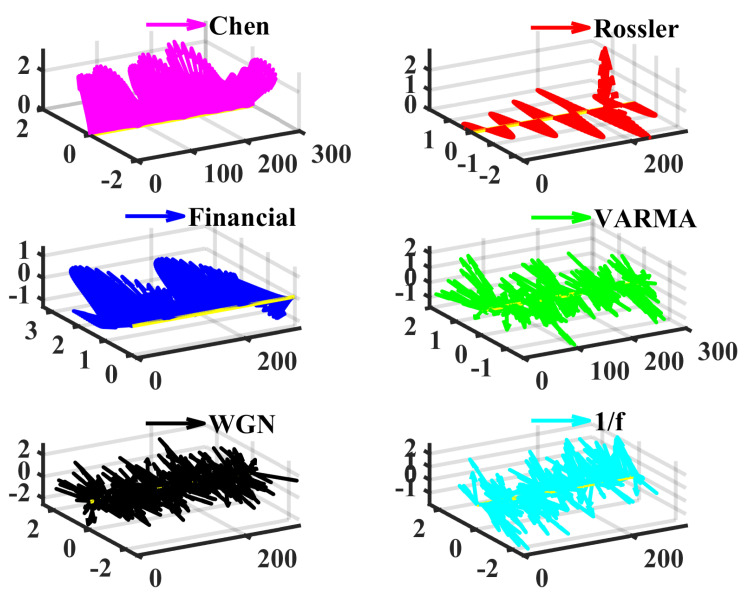
Examples of chaotic and stochastic series. The time step is 0.005 for the Chen system, and 0.05 for the Rössler and financial systems. Compare to Figure 2, the Rössler and financial series show significant oscillations.

However, one problem is that $LSE^{rossler}$ and $LSE^{financial}$ cannot be distinguished, as they are coincident in Figure 4. The reason for this is that the simulated series of Equations (12) and (13) is short and the time step is too small, thus they cannot reflect the feature of the whole system. So, we change the time step to 0.05 and the time span to [0:0.05:300] for the above two systems in order to verify whether LSE functions or not. Examples of the above six kinds of signals are shown in Figure 5.

**Figure 6:**
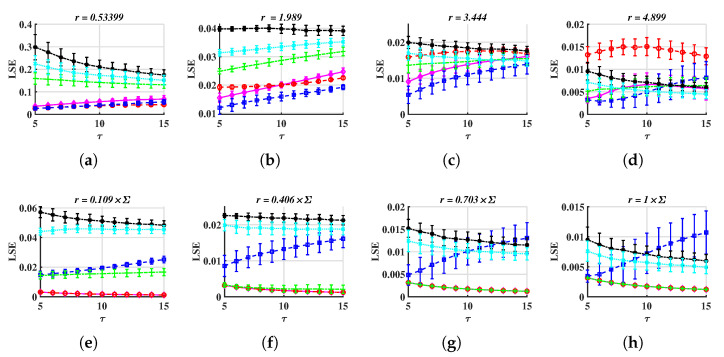
LSE values, as functions of τ for several fixed tolerances or coefficients. The time step is 0.05 for the Rössler and Financial systems and 0.005 for the Chen system. (**a**–**d**) METHOD A, (**e**–**h**) METHOD B.

After repeating 100 times, similar to what we have done in previous tasks, we perceive that LSE works for many tolerance values and some results are drawn in Figure 6. Here, we just show several LSE-τ figures for fixed tolerances or coefficients, such as Figure 4e,n, because LSE-*r* charts appear too similar to disparate different series.

From Figure 6, one can easily distinguish six series. For example, in Figure 6d, $LSE_{A}^{rossler}$ lies at the top, in Figure 6b, the LSE values of the other five series do not intersect, and the order is WGN, 1/f noise, VARMA, Chen, and financial, from the top to the bottom. However, the coefficient should be small, such as it is around 0.4 (Figure 6b,f). Otherwise, LSE curves are overlapped (Figure 6d,h).

Moreover, to test the dependence on the distance function, we replace Equation (3) with the distance derived from the $l_{\infty }$ norm, i.e.,

(15) $$dis_{j}=|y_{0}^{i},y_{1+j}^{i}{|}_{\infty }=max\left|y_{0}^{i}-y_{1+j}^{i}\right|$$

**Figure 7:**
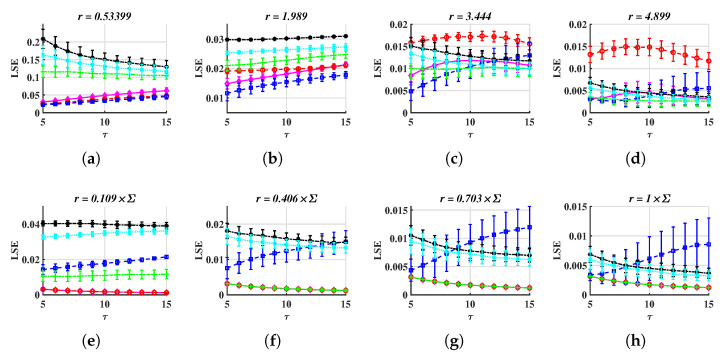
LSE values, where the distance function is derived from the infinite norm, Equation (15). (**a**–**d**) METHOD A, (**e**–**h**) METHOD B.

Repeat the above simulations, the corresponding results are drawn in Figure 7.

Compare Figure 7 to Figure 6, one can see that LSE with the same range of parameters can still effectively distinguish between different signals when using the $l_{\infty }$ norm-derived distance, as Figure 7a,b,f show. Meanwhile, the range of *r* should be carefully chosen, or the LSE curve may be overlapped, such as Figure 7d,h.

**Figure 8:**
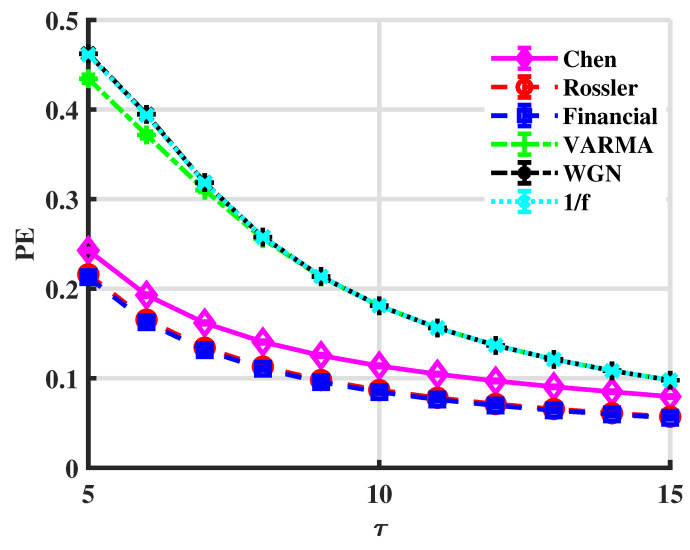
PE values. The embedding dimensions are {5,6,…,15}.

To compare with the multivariate permutation entropy (PE) [17,58], Figure 8 shows PE curves for the above six series.

Chaotic signals can be distinguished from stochastic ones via PE since $PE^{chaos}<PE^{random}$ for all τ in Figure 8. However, PE cannot discriminate random series, which have correlations in different degrees. Moreover, it fails in the Rössler and financial cases. It means that PE is not sensitive to tiny differences in the complexity of systems. However, LSE can distinguish those systems, which indicates LSE is a more accurate measure of complexity.

### 3.2. Integer-Order Chaos, Fractional-Order Chaos, and Random Series

In this subsection, we test LSE on the integer-order chaotic systems, fractional-order chaotic systems, and stochastic sequences. For integer-order chaos, we use Chen, Rössler, and financial systems. The timespans are 0:0:005:30, 0:0.05:300, and 0:0.05:300, respectively. In each simulation, small disturbances are added to the initial values. See Equations (16) to (18).

1. Integer-order Chen system [52]
  (16) $$\left\{\begin{array}{c} \dot{x}=a\left(y-x\right)\\ \dot{y}=dx-xz+cy\\ \dot{z}=xy-bz \end{array}\right.$$
  a=35, b=3, c=28, d=c−a=−7, initial values $\left(x_{0},y_{0},z_{0}\right)=\left(-9,-5,14\right)+\epsilon$.
2. Integer-order Rössler system [53]
  (17) $$\left\{\begin{array}{c} \dot{x}=y-z\\ \dot{y}=x+ay\\ \dot{z}=b+z\left(x-c\right) \end{array}\right.$$
  a=0.4, b=2, c=4, $\left(x_{0},y_{0},z_{0}\right)=\left(0.5,1.5,0.1\right)+\epsilon$.
3. Integer-order financial system [54]
  (18) $$\left\{\begin{array}{c} \dot{x}=z+\left(y-a\right)x\\ \dot{y}=1-by-x^{2}\\ \dot{z}=-x-cz \end{array}\right.$$
  a=1, b=0.1, c=1, $\left(x_{0},y_{0},z_{0}\right)=\left(2,-1,1\right)+\epsilon$.

With the given parameters, the above three systems produce three-dimensional chaotic series.

For fractional-order chaos, Equations (11) to (13) are used to generate chaotic time series, such as in Section 3.1. Additionally, we add a small disturbance ($Disturb_{\alpha }=\pm 0.02\times \epsilon$) to parameters ($\alpha_{1}$, $\alpha_{2}$, and $\alpha_{3}$), in each simulation. Moreover, we use the same methods as in Section 3.1 to generate random signals. All the simulated series have the same size (500 × 3).

**Figure 9:**
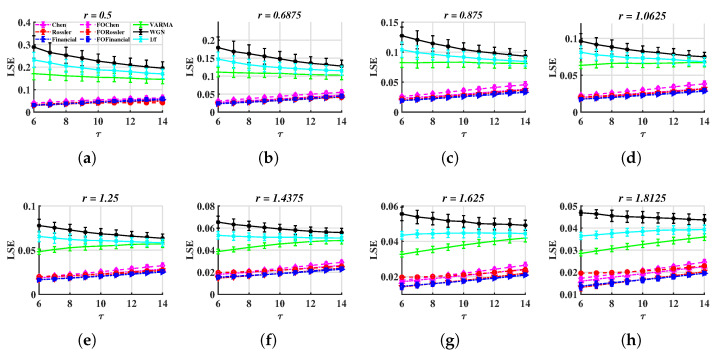
LSE values with τ∈{6,7,…,14} and different tolerances. (**a**–**d**) r= 0.5, 0.6875, 0.8750, 1.0625, (**e**–**h**) r=1.2500, 1.4375, 1.6250, 1.8125.

Since we aim to illustrate the effectiveness of LSE, here, we only employ METHOD A to distinguish between different types of signals. Similar to Section 3.1, we also repeat the simulation procedure 100 times; τ∈{6,7,…,14} and r∈[0.5,1.81]. The results are drawn in Figure 9.

In Figure 9, chaotic signals can be distinguished from random ones. For instance, $LSE^{random}$ is greater than 0.1 but $LSE^{chaos}$ is less than 0.1 in Figure 9a. A similar situation can be seen in the images corresponding to other *r* values.

**Figure 10:**
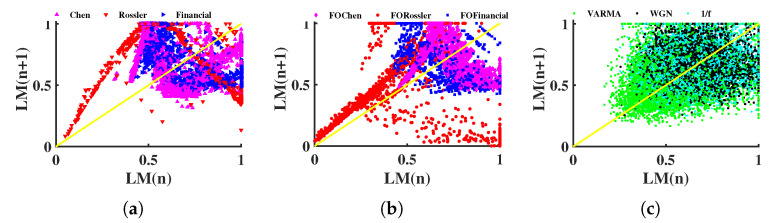
Return map. LM presents the local maxima of each length sequence, which is mapped into [0, 1] by the min–max method. (**a**) Integer-order chaotic systems, (**b**) fractional-order chaotic systems, (**c**) random signals.

The return map (RM) can be used to characterize the nonlinear process by the transformation of the local maxima (or minima) of the signal [59,60,61,62]. We tested RM on the length sequences associated with the above nine multivariate time series and the results are drawn in Figure 10.

The difference between the Rössler system and the other signals is relatively obvious in both the integer-order and fractional-order cases, as shown in Figure 10a,b. However, it is difficult to distinguish between the Chen system and financial system. In addition, both the Chen and Financial systems are mostly located in [0.5,1]×[0.5,1], overlapping with the position where random signals are located, see Figure 10c. Thus, Figure 10 shows that several signals are muddled and difficult to differentiate apart. This failure could be related to the time series’ length, since RM commonly calls for a sizable amount of data points.

Three shortcomings noted in Section 1 have been somewhat mitigated by the LSE method.

1. Multi-scale entropy and complex networks often necessitate a substantial number of data nodes to obtain useful findings, but LSE can process relatively short time series. The length of the test cases in this section is only 500.
2. According to the calculation process in Section 2, it is simple to know that the time complexity of the LSE method is linear (O(τn)) and appropriate for handling real-time jobs.
3. LSE is more effective at depicting the short-term autocorrelation and the correlation between various components of multidimensional time series because it takes advantage of the similarity of vectors in sliding windows.

**Figure 11:**
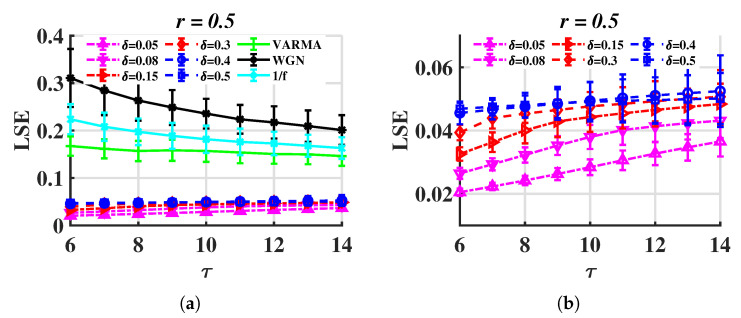
LSE for trajectories of the Rössler system with different time step sizes (δ). (**a**) $LSE^{rossler}$ for different time step sizes and $LSE^{random}$, (**b**) $LSE^{rossler}$ only.

In Section 3.1, the time steps for Rössler and financial systems are 0.005 and 0.05, and the LSE values vary remarkably. Here, we test LSE on the Rössler system with different steps. The results are shown in Figure 11. It can be seen that the increase in the step length causes LSE to increase, but the gap between $LSE^{rossler}$ and $LSE^{random}$ is still clear.

**Figure 12:**
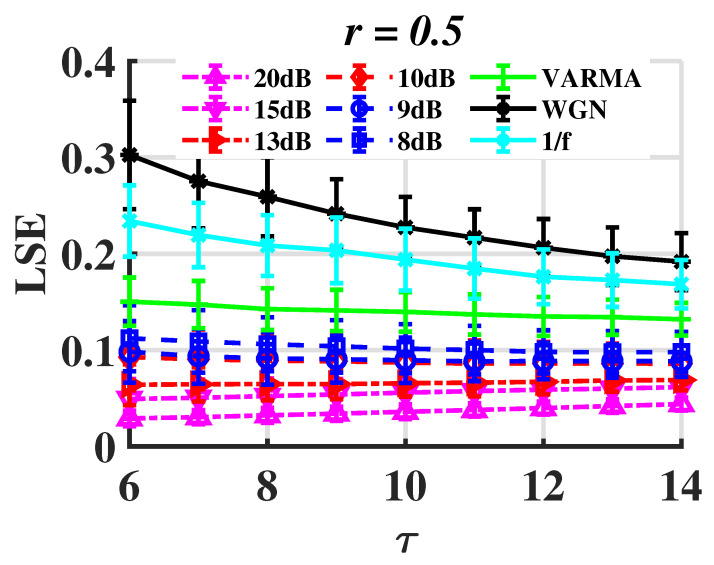
LSE of the Rössler system with WGN.

In order to assess the robustness of the proposed method, the original signal is supplemented with Gaussian white noise at different levels, and the LSE values are recorded accordingly. The original time series is produced by the chaotic Rössler system. The signal-to-noise ratio (SNR) is used to measure the level of background noise, which is defined by

(19) $$SNR=10\times log_{10}\left(\frac{P_{S}}{P_{N}}\right)$$

where $P_{S}$ is the power of the signal and $P_{N}$ is that of noise. See Figure 12.

Noise tolerance is present in LSE to some extent. The $LSE^{rossler}$ is less than 0.1 when the SNR is higher than 10 dB. As a result, the random signals differ significantly. LSE, however, is unable to properly discriminate between chaotic and random signals as SNR continues to drop.

The above analysis indicates that the proposed LSE, based on its variations in different scales for certain tolerances (or coefficients), can be regarded as an efficient tool to identify multivariate time series. In the next section, we attempt to apply LSE to real-world data, such as the financial market index and machinery data.

## 4. Application on Real-World Data

As we introduced in Section 1, multivariate time series conceal the characters in the autocorrelation of each component, as well as the cross-correlation between some channels, which makes it difficult to extract suitable features for further exploration. Two real-world applications, one for the financial market and another for fault diagnosing, will be discussed below.

### 4.1. Financial Market Index

**Figure 13:**
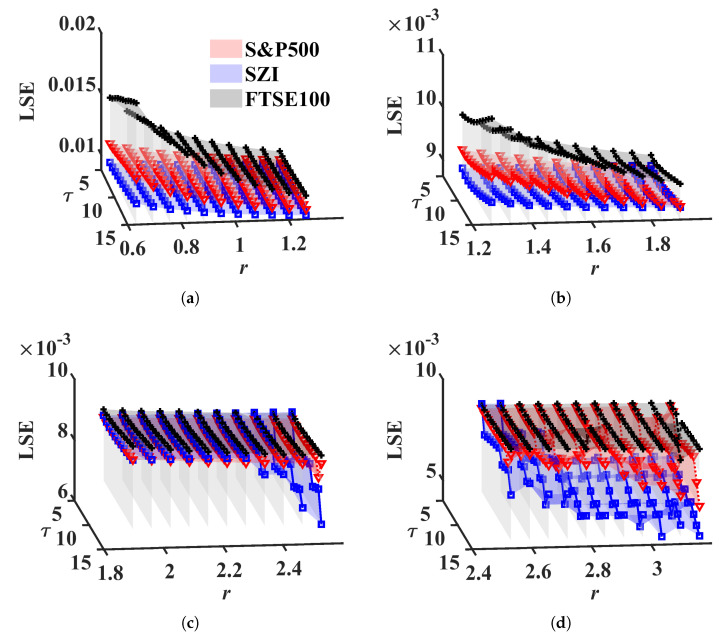
LSE values for stock market index. (**a**) r=[0.6325:0.6325:1.2649] (Start:time step:End), (**b**) r=[1.2649:0.6325:1.8974], (**c**) r=[1.8974:0.6325:2.5298], (**d**) r=[2.5298:0.6325:3.1623].

Financial time series are typical signals with high complexities. How do we illustrate the discrimination between financial markets in different regions? Here, we use LSE to quantify the complexities of three important indices, S&P500, FTSE100, and Shenzhen Securities Component Index (SZI). Their values can be attained from Yahoo.com [63]. The time period is between 25/06/2017 and 24/06/2022. In this experiment, daily OHLC (open, high, low, close) prices and volume are considered. In order to avoid the result being manipulated by the volume only, METHOD A is employed to explore the complexity of the three indices, because volume values have much higher standard deviations than OHLC prices. For more details, see Table 1. The scales are {5,6,…,15} and the tolerance is between 0.63 and 3.16. The results are drawn in Figure 13.

From Figure 13, we can see that $LSE^{FTSE100}$ lies at the top, $LSE^{SZI}$ at the bottom, and $LSE^{S\&P500}$ is between them when r<1.8. That is, the European stock market shows higher complexity than the other two markets; maturate financial markets, European, and American stock markets display more complexity and stability than the Chinese stock market, which is in accordance with Cao [64]. Note that $LSE^{SZI}$ lies between $LSE^{FTSE100}$ and $LSE^{S\&P500}$ for 1.8<r<2.4. It contradicts the above result. The reason for this can be attributed to the value of *r*. We are aware that the range *r* is determined by multi-dimensional WGN, but since the financial market index actually has a significant correlation, the tolerance should be lower than usual.

### 4.2. Machinery Fault Recognition

**Figure 14:**
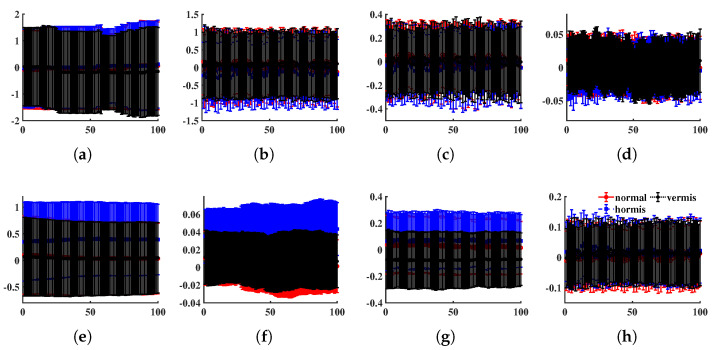
The average and standard deviation of each component (first 100 observations), where the *x*-axis represents time and the *y*-axis the readings. (**a**) Tachometer signal, (**b**–**d**) underhang bearing accelerometer: axial, radial, and tangential directions, (**e**–**g**) overhang bearing accelerometer: axial, radial, and tangential directions, (**h**) microphone.

In this part, we examine the ability of the LSE to recognize vibration signals produced by normal or faulty mechanical systems. The bearing dataset is from the machinery fault database (MAFAULDA), which is kindly provided by the Signals Multimedia and Telecommunications Laboratory (SMT) of the Federal University of Rio de Janeiro (UFRJ) [65]. MAFAULDA collects multivariate time series recorded by sensors on a SpectraQuest’s machinery fault simulator (MFS) alignment–balance–vibration (ABVT) and comprises six different simulated states. We tested LSE for three states: normal function as well as horizontal and vertical misalignment faults. The data acquisition system is composed of several sensors: one Monarch Instrument MT-190 analog tachometer, three Industrial IMI Sensors accelerometers (Model 601A01), one IMI sensors triaxial accelerometer (model 604b31), and a Shure SM81 microphone. Each sequence has 8 columns sampled at 50 KHz for 5 s, namely a 250,000 × 8 matrix. We randomly intercept 100 fragments (each has 3000 rows) for every state from the database as the test dataset. Figure 14 shows the distribution of each component of the test data (first 100 rows).

**Figure 15:**
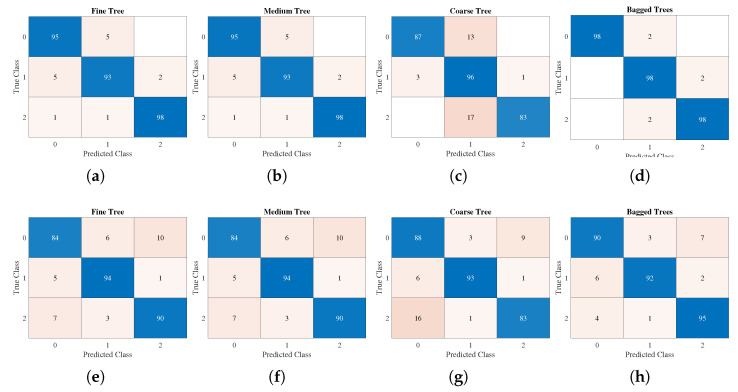
Confusion matrix. Class0–normal, class1–horizontal misalignment, class2–vertical misalignment. (**a**–**d**) LSE, (**e**–**h**) PE.

Then, we employed METHOD A to compute the LSE values. The scale set is {5,6,…,15} and the tolerance is 0.2457. Moreover, the decision tree method is applied to classify LSE values of different states. A decision support tool known as a decision tree employs a tree-like paradigm to represent options and their outcomes. It consists of nodes, branches, and leaves, each of which displays a property, a rule, or an outcome [66]. In this study, three alternative classification algorithms based on the decision tree theory, fine tree (about 100 leaves make fine distinctions between classes), medium tree (less than 20 leaves with medium flexibility), and coarse tree (less than 4 splits), were used to categorize the vibration signals [67]. In addition, an ensemble bagging tree classifier was added, which is processed by creating numerous decision trees during training and outputs the majority of these tree choices for classification tasks [68]. The ten-fold cross-validation was employed in this test and the confusion matrix was plotted in Figure 15a–d. Nevertheless, if we replace LSE by PE, the accuracy is significantly lower; see Figure 15e–h.

From Figure 15, bagged trees based on LSE had the highest accuracy at 98%, while that of PE was 92.3%. Moreover, the accuracies of the LSE-based fine tree, medium tree, and coarse tree (95.3%, 95.3%, and 88.7%) were higher than those of PE (89.3%, 89.3%, 88%). In short, as the LSE values (as features) contribute higher accuracies than PE, the proposed LSE can be an efficient tool for distinguishing multivariate real-world time series.

## 5. Conclusions

In this paper, we proposed a local structure-based entropy (LSE), which reflects recurrence conditions in certain scales. It can be regarded as an index of complexity for multivariate time series. Depending on whether or not the components are normalized, we suggest two strategies for using LSE: one shows greater discrimination while the other is more stable but easily ignores the effects of slightly varying components. When the tolerance is small, LSE values of fractional chaotic time series are significantly lower than those of stochastic ones. Moreover, the LSE method also has some resilience to noise. When the SNR is higher than 10 dB, accurate classification is obtained for the task of differentiating chaotic signals from random ones, but the accuracy declines when SNR drops. With suitable tolerance, LSE (in certain scales) can be considered a feature of a dynamical system. Regarding real-world data, it was applied to a financial market index, indicating that European and American financial markets are more complex and stable than Chinese markets. Furthermore, we tested LSE on MAFAULDA; it resulted in a higher accuracy than PE-based classification.

The results are sensitive to the parameters (especially for *r*), but the optimal range is provided by the simulation other than the theoretical calculation. Therefore, a more comprehensive examination of the parameters and LSE values based on basic information, such as dimensionality, correlation, and time series length, is required. A distance function that can eliminate the impact of the correlation can also be utilized to increase the applicability of LSE.

## Figures and Tables

**Table 1:** Financial market index information.

Index	Length	Region
S&P500	1256	American
SZI	1220	China
FTSE 100	1263	Europe

## Data Availability

MAFAULDA, in Section 4.2, is available at http://www02.smt.ufrj.br/~offshore/mfs/page_01.html, and we have accessed on 15 July 2022.

## Appendix A

### Appendix A.1

In **NOTE 1**, we suggest that the range of tolerance *r* can be chosen by referring to the standard normal distribution. In fact, we adopt this strategy in METHOD A. For two n-dimensional random variables whose components are N(0,1) iid, Proposition A1 shows the variance of the square of the Euclidean distance is 8n.

**Proposition** **A1.**
*Assume $x_{i}∼N\left(0,1\right)$ and $y_{i}∼N\left(0,1\right)$ are iid, let $SS=\Sigma_{i=1}^{n}{\left(x_{i}-y_{i}\right)}^{2}$, then D(SS)=8n.*

**Proof.** Let $z_{i}=\frac{x_{i}-y_{i}}{\sqrt{2}}$, then $z_{i}∼N\left(0,1\right)$. $S=\Sigma_{i=1}^{n}{\left(\frac{x_{i}-y_{i}}{\sqrt{2}}\right)}^{2}=\Sigma_{i=1}^{n}{\left(z_{i}\right)}^{2}∼\chi^{2}\left(n\right)$, then D(S)=2n. Since $S=\frac{1}{2}SS$, we have D(SS)=D(2S)=8n, thus $\sigma \left(SS\right)=2\sqrt{2n}$. □

For METHOD A in Section 3, the scale is τ∈[5:1:15]; thus, the set of *r*, denoted by *R*, is defined as

(A1) $$R=\left[0.05:\frac{0.5-0.05}{11}:0.5\right]\times \sqrt{8\times n}$$

*R* can be regarded as a sequence of threshold values multiplied by a constant. Each element of this sequence ([·:·:·] part) is called a coefficient.

The standardization is required for METHOD A, but it can shuffle the positions of the observed data. To overcome this drawback, METHOD B is introduced. Instead of standardization, different ranges of *r* are utilized to fit different time series. These ranges are selected according to the variance of the observed time series. In fact, *R* is defined as

(A2) $$R=[0.05:\frac{0.5-0.05}{11}:0.5]\times \Sigma$$

where Σ is introduced in Proposition A2.

### Appendix A.2

**Proposition** **A2.**
*Let $x={\left(x_{i}\right)}_{i\leq n}$, where $x_{i}∼N\left(\mu_{i},\sigma_{i}^{2}\right)$, be a n-dimensional random variable and assume its components are independent; x and y are iid. Denote $SS=\sum_{i=1}^{n}{\left(x_{i}-y_{i}\right)}^{2}$, then $\Sigma =D\left(SS\right)=8\sum_{i=1}^{n}\sigma_{i}^{4}$.*

**Proof.** Assume $X∼N(\mu ,\sigma^{2})$. It is easy to know that $E\left(X^{2}\right)=\sigma^{2}+\mu^{2}$ and $E\left(X^{4}\right)=\mu^{4}+6\mu^{2}\sigma^{2}+3\sigma^{4}$. Then,

(A3) $$\begin{array}{cc} D(X^{2}) & =E\left[{\left(X^{2}\right)}^{2}\right]-{\left[E\left(X^{2}\right)\right]}^{2}\\ \; & =\mu^{4}+6\mu^{2}\sigma^{2}+3\sigma^{4}-{\left(\sigma^{2}+\mu^{2}\right)}^{2}\\ \; & =4\mu^{2}\sigma^{2}+2\sigma^{4} \end{array}$$

Note that $\left(x_{i}-y_{i}\right)∼N\left(0,2\sigma_{i}^{2}\right)$, by Equation (A3) we have $D\left[{\left(x_{i}-y_{i}\right)}^{2}\right]=8\sigma_{i}^{4}$. Then,

(A4) $$\begin{array}{cc} D(SS) & =D(\sum_{i=1}^{n}{\left(x_{i}-y_{i}\right)}^{2})\\ \; & =\sum_{i=1}^{n}D\left[{\left(x_{i}-y_{i}\right)}^{2}\right]\\ \; & =8\sum_{i=1}^{n}\sigma_{i}^{4} \end{array}$$

□
